# Incorporating Scale Dependence in Disease Burden Estimates: The Case of Human African Trypanosomiasis in Uganda

**DOI:** 10.1371/journal.pntd.0002704

**Published:** 2014-02-13

**Authors:** Finola Hackett, Lea Berrang Ford, Eric Fèvre, Pere Simarro

**Affiliations:** 1 Department of Geography, McGill University, Montreal, Quebec, Canada; 2 Veterinary and Infectious Diseases, Institute of Infection and Global Health, University of Liverpool, Neston, United Kingdom; 3 Department of Control of Neglected Tropical Diseases, World Health Organization, Geneva, Switzerland; Foundation for Innovative New Diagnostics, Switzerland

## Abstract

**Background:**

The WHO has established the disability-adjusted life year (DALY) as a metric for measuring the burden of human disease and injury globally. However, most DALY estimates have been calculated as national totals. We mapped spatial variation in the burden of human African trypanosomiasis (HAT) in Uganda for the years 2000–2009. This represents the first geographically delimited estimation of HAT disease burden at the sub-country scale.

**Methodology/Principal Findings:**

Disability-adjusted life-year (DALY) totals for HAT were estimated based on modelled age and mortality distributions, mapped using Geographic Information Systems (GIS) software, and summarised by parish and district. While the national total burden of HAT is low relative to other conditions, high-impact districts in Uganda had DALY rates comparable to the national burden rates for major infectious diseases. The calculated average national DALY rate for 2000–2009 was 486.3 DALYs/100 000 persons/year, whereas three districts afflicted by rhodesiense HAT in southeastern Uganda had burden rates above 5000 DALYs/100 000 persons/year, comparable to national GBD 2004 average burden rates for malaria and HIV/AIDS.

**Conclusions/Significance:**

These results provide updated and improved estimates of HAT burden across Uganda, taking into account sensitivity to under-reporting. Our results highlight the critical importance of spatial scale in disease burden analyses. National aggregations of disease burden have resulted in an implied bias against highly focal diseases for which geographically targeted interventions may be feasible and cost-effective. This has significant implications for the use of DALY estimates to prioritize disease interventions and inform cost-benefit analyses.

## Author Summary

Since the 1990s the World Health Organisation has established the disability-adjusted life year (DALY) as a metric for the burden of human disease and injury. However, disease burden has primarily been estimated at the national scale, which does not account for sub-country variations in burden levels. We used the case of human African trypanosomiasis (HAT), a highly focal NTD, in Uganda to calculate and map burden in DALYs. Our results show that HAT burden is highly sensitive to under-reporting estimates, and is particularly high in heavily affected parishes and districts of Uganda. Some districts in southeastern Uganda had HAT burden rates comparable to the national burden rates of major infectious diseases such as malaria and HIV/AIDS. Thus, the spatial scale of burden estimation is crucial, especially for focal diseases such as HAT, and national-level estimates may not reflect the level of impact in afflicted communities. We recommend sub-country burden estimation to identify key areas for prioritization of disease surveillance and targeted interventions.

## Introduction

Developed by the World Health Organization (WHO) in the early 1990s as part of the creation of the Global Burden of Disease Study (GBD), the Disability Adjusted Life Year (DALY) is a standardized metric that has been utilized widely in the two decades since to estimate the global and regional impact of diseases, set health care priorities, and assess the cost-effectiveness of targeted interventions. The publication of the first GBD study established the DALY as a standard metric for evaluating health outcomes and facilitating comparison between regions, diseases, and types of burden [1], [2]. Comprehensive global burden assessments have been published regularly, providing a temporal lens on changing global health burden; the initial report in 1990 was followed by periodic publications throughout the past two decades, with the 2010 GBD study most recently published in late 2012, and involving significant methodological changes compared to previous iterations.

The DALY as a global health measure has not been without controversy, however. Of particular critique have been burden assessments for neglected tropical diseases (NTDs), predominantly in sub-Saharan Africa (SSA). In 2010, GBD estimates placed the total burden of NTDs at about 108.7 million DALYs or 4.4% of the total global burden of disease and injury [3]. This represents a significant increase from the GBD estimate in 2002 of 20 million DALYs or 1.3% of the total global burden of disease and injury [4]. Other estimates have found that overall, NTDs account for up to 56.6 million DALYs globally, and 8.6–21.2 million DALYs in SSA [5]. These estimates are subject to high uncertainty, however, given a lack of adequate disease data for most NTDs in SSA [6]. The socio-economic impact of NTDs, encompassing various parasitic and bacterial infections affecting largely poor populations in tropical regions, has been poorly accounted for by DALY calculations [7], [8]. Despite the value of GBD metrics, therefore, the utility of DALY calculations for NTDs remains problematic. This is particularly true for zoonotic NTDs which impose a dual burden through human illness as well as economic losses to livestock industries [9]. These limits in assessing human disease burden for NTDs have been acknowledged with the publication of the first WHO report on NTDs [10].

Human African trypanosomiasis (HAT), or sleeping sickness, is a parasitic, zoonotic, vector-borne infection endemic to SSA. HAT causes non-specific febrile illness in the early stage, with progression to neuropsychiatric disturbances in the late stage, resulting in coma and death if untreated [11]. HAT primarily affects remote rural communities in SSA. The acute, *rhodesiense* form of the parasite is confined to eastern SSA, while the chronic, *gambiense* form occurs in western and central SSA [12]. HAT has a severe impact on affected communities as it causes not only social and economic losses due to human illness, but also economic losses from livestock morbidity and mortality caused by the zoonotic form of the infection [13]. Omission of these indirect impacts also have implications for prioritization of funding and cost-benefit estimates for intervention programs. In addition, control measures – for *rhodesiense* HAT in particular – need to account for the dual impact of human and livestock trypanosomosis prevalence [14]. The global incidence of HAT has been estimated at 17 500 cases annually, and 50 000–70 000 cases from 2000–2005 [15]. Current GBD estimates show a significant decrease in recent years, from 1.82 million DALYs attributed to HAT globally in 2000, to 829 000 DALYs in 2005 and 560 000 DALYs in 2010 [3]. However, as a zoonotic NTD with highly focal distributions across the continent, HAT has been difficult to adequately estimate within the GBD framework. Furthermore, Simarro et al. [16] highlighted the risks of reduced HAT elimination efforts as a result of lowered national burden estimates, arguing that burden-driven decreases in funding for HAT surveillance and control could lead to disease re-emergence.

Focal or epidemic-prone diseases present a particular challenge for burden calculation. Uncertainty exists in estimating the populations at risk, and the use of large geographic units for assessment poorly reflects spatial heterogeneity of incidence [4], [5]. HAT is a focally concentrated disease, occurring in endemic foci within tsetse-infested zones of SSA, and *gambiense* HAT in particular shows significant spatial variation at the micro level [17]. Disease mapping and spatial analysis has thus long been a crucial component of HAT surveillance, prevention, and treatment [12]. Due to its focal nature, HAT imposes a particularly high burden on affected communities, yet ranks poorly in national or regional burden assessments [18]. The spatial scale for burden assessments is thus critical to comparative estimates of burden and assessment of intervention [19]. HAT incidence varies significantly in time as well as space. In Uganda, HAT incidence decreased over the 2000–2009 study period, from 948 cases of *gambiense* HAT in 2000 to 99 new cases in 2009. *Rhodesiense* HAT showed a similar decrease, from 300 new cases in 2000, peaking at 473 in 2005, to 129 cases in 2009. The specific incidence of these cases in areas of Uganda has shifted over time, for instance, with the movement of *rhodesiense* foci north into central Uganda, approaching the *gambiense*-endemic area [12]. Temporal variation in HAT must thus also be considered when examining the spatial distribution of the disease. With some 60–95% of HAT cases unreported [20]–[22], and mortality 100% for untreated patients [23], HAT burden – like many neglected tropical diseases – is inadequately represented within the country-based GBD framework.

The GBD study assessments are based on nationally aggregated estimates. Efforts to estimate focal HAT burden have focused on the community level [24]–[26]. At the regional level, Odiit et al. [27] assessed the burden of *rhodesiense* HAT across Africa and in southeastern Uganda. While such studies provide valuable estimates of local burden in highly affected regions, no studies have yet estimated sub-national burden for an entire country. National level estimates are critical to inform broader policy-making and prioritization of interventions. Here we present the first study to assess spatially-disaggregated HAT burden at the national level. We focus on Uganda, the only country where both *rhodesiense* and *gambiense* HAT are endemic, and where concerns of parasite species convergence have raised international concern and emergency intervention [28]–[30]. The temporal variation in HAT incidence in Uganda shows an overall decrease from 2000–2009. 948 new cases of *gambiense* HAT were reported in 2000 compared to 99 in 2009; similarly, 300 cases of *rhodesiense* HAT were reported in 2000, peaking at 473 in 2005, and decreasing to 129 in 2009. Notwithstanding this decreasing trend, due to the significant spatial variation in incidence, specific areas of Uganda have seen HAT foci reduction, emergence, or resurgence over this time period [12].

**Figure 1:**
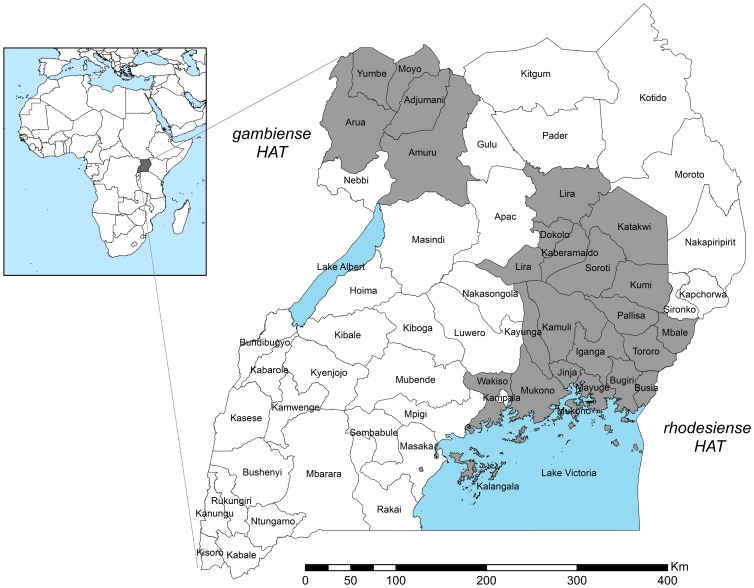
Study area. Shaded districts in Uganda affected by HAT over the study period, 2000–2009.

The objectives of this study were to estimate the burden in DALYs of *rhodesiense* and *gambiense* HAT in Uganda for 2000–2009 on the sub-national scale, accounting for both reported and unreported cases, and to map the results by parish and by district to compare the spatial distribution of burden. Figure 1 shows the study area and districts affected by HAT incidence during the study period.

## Methods

### DALY definition

The disability-adjusted life year (DALY) is a summary health measure which incorporates both mortality and morbidity, the former in terms of years of life lost (YLLs) and the latter in years of life lived with disability (YLDs). Premature mortality is evaluated based on the age-distribution of mortality attributable to each condition, using standard model life expectancies. For YLDs, a disability weighting between 0 and 1, where 0 is equivalent to perfect health and 1 is equivalent to premature death, is assigned to a condition to quantify time lived with a disability based on its severity. When multiplied by incidence and duration of a particular disease or condition, this makes debilitation as a result of disease or injury comparable with mortality estimates. Thus, YLLs and YLDs are summed to estimate total DALYs. Burden is generally discounted to represent societal time preference and age-weighted to account for different social roles at different ages. Finally, DALYs are aggregated at the national level and presented as summed estimates for each disease or condition present in a particular country. [31]

### GBD framework and HAT

Prior to the GBD 2010 study, GBD DALY estimates used standard West Level 26 life tables with a female life expectancy at birth of 82.5 years, a time discount rate of 3% and an age-weighting function 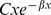 with parameters *C* = 0.1658 and *β* = 0.04 [31]. The recently published GBD 2010 study uses a new standard life table with a life expectancy at birth of 86.0 years, accounts for comorbidity in calculating YLDs, and does not discount DALYs over time or employ age weighting [32]. The revised GBD 2010 calculations estimated a total burden of 168 651 DALYs for HAT in Uganda in 2000, 97 041 DALYs in 2005, and 29 079 DALYs in 2010 [3]. Here, we use the pre-2010 GBD parameters and estimates as the GBD 2010 methods and results were not fully published at the time of this study.

The 2004 GBD study estimated a total HAT incidence in SSA of 60 300, combining separate estimates of *rhodesiense* and *gambiense* HAT incidence in which “completeness of reporting of cases was assumed to be around 33% for *T.b. gambiense* and 5% for *T.b. rhodesiense*” [22]. Published DALY calculations for HAT burden use a disability weighting of 0.191 for both *rhodesiense* and *gambiense* HAT, and do not distinguish between stages of disease [19]. Furthermore, the GBD 2004 study assumed durations of 5 years for *gambiense* HAT and 1 year for *rhodesiense* HAT. Lastly, the GBD 2004 assumed 85% of cases were untreated with 100% case fatality, and 15% treated with 5% case fatality. GBD calculations based on these parameters resulted in a total estimated burden of 59 423 DALYs for HAT in Uganda in 2004 [22]. This is notably less than the GBD 2010 estimate of 97 041 DALYs for 2005, thus, estimates using the pre-2010 parameters may be considered conservative.

### DALY formula and parameterization

Burden was calculated using the standard DALY formula as published in the Global Burden of Disease framework, with several important modifications (Table 1). We estimated burden using the DALY formula for an individual:
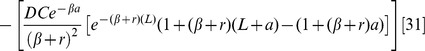
DALYs were calculated using the standard discount rate of 3% (*r* = 0.03), with and without age-weighting (*C* = 0.1658 and *β* = 0.04). Since population-specific life tables are appropriate for regional burden studies, Uganda-specific life tables were used to provide a regionally specific estimate of burden due to premature mortality [19], [33]. We acknowledge that this would bias our results towards lower burden estimates relative to GBD DALY totals, which use standard model life expectancies. However, given that Uganda life table values were significantly below model expectancies, their use ensured locally representative and conservative burden estimates. We additionally used HAT-specific parameters to provide for more accurate burden estimation. See Table 1 for a full summary of parameters used in DALY calculations. As proposed by Fèvre et al. [19], these included parasite- and stage-specific disability weightings, durations, and mortality rates, in recognition that the *rhodesiense* and *gambiense* forms of HAT, as well as the early and late stages, have significantly different clinical manifestations and rates of disease progression [11]. In order to allow spatially disaggregated burden estimates, DALY burden was calculated for individual cases. The GBD study calculates burden based on national incidence totals; DALY estimation for HAT on a spatially disaggregated sub-national scale has not previously been calculated.

**Table 1:** Summary of parameters used in DALY formula.

Parameter	DALY formula	Value(s)	Comments	Source(s)
**Age (a)**	YLLs and YLDs	Assigned based on negative binomial function	Assumed age distribution of *gambiense* HAT cases equal to *rhodesiense* HAT cases	[36], see Figures S1 and S2
**Duration of illness (L)**	YLDs	*rhodesiense* HAT: 0.25 (early), 0.25 (late); *gambiense* HAT: 2 (early), 1 (late)	Estimated based on multiple sources; did not account for variability in disease duration or early treatment and diagnosis	*rhodesiense* HAT: [39], [41], [43]; *gambiense* HAT: [24], [40]
**Disability weight (D)**	YLDs	*rhodesiense* HAT: 0.21 (early), 0.81 (late); *gambiense* HAT: 0.191 (early), 0.81 (late)	Stage-specific weights have not been derived for *gambiense* HAT; assumed similar to late-stage *rhodesiense* HAT	[22], [25]
**Mortality**	YLLs	Assigned based on quadratic function, adjusted for average mortality rates of: *rhodesiense* HAT: 2.4% (early), 8.1% (late); *gambiense* HAT: 1% (early), 3% (late)	Broadly conservative estimates, to account for varying mortality of different HAT treatment regimens	[36], [44], see Figure S3
**Life expectancy (L)**	YLLs	Uganda life tables	Assumed rate of life expectancy increase in Uganda relatively steady from 2000–2009; accounted for case sex ratio	[37], see Table S1

Data for 2846 cases of *rhodesiense* HAT and 4026 cases of *gambiense* HAT in Uganda were obtained from the WHO Atlas of Human African Trypanosomiasis [12]. The Atlas has been used to estimate the population at risk for HAT in SSA [34], [35], and provides standardized and collated data for spatially and temporally explicit HAT cases across the continent. Atlas data was collected on historic case incidence for the years 2000–2009. We note that the incidence of new cases of both *gambiense* and *rhodesiense* HAT declined over this time period [12]. Thus, our burden estimates reflect historic values and are not meant to portray the current situation of HAT in Uganda. DALY calculations were run 100 times, with the modelled case age and mortality randomly assigned in each iteration. Age data were not available, so age was randomly assigned based on an existing age distribution of HAT cases drawn from a sample of cases from southeastern Uganda [36]. Distribution fitting was conducted using STATA (StatCorp version 11). The stage-specific age distributions were modelled with a negative binomial function, and age was randomly assigned in the DALY model (see Figures S1 and S2). Uganda-specific life tables were obtained from the WHO [37]. Life expectancy values for both sexes for 2000 and 2009 were averaged and considered representative of the 10-year time period (see Table S1). This assumed minimal bias since life expectancy in Uganda increased fairly steadily over the study period [37]. Life expectancy also incorporated the sex ratio in *rhodesiense* HAT case incidence where 51.6% of cases were male [36]. Stage categorisation was available for the majority of reported HAT cases (see Table 2 for a breakdown). Cases with no stage data available were considered as early-stage in the DALY calculations, so as to conservatively estimate burden.

**Table 2:** Burden of reported HAT cases in Uganda, 2000–2009.

	*Rhodesiense* HAT	*Gambiense* HAT
Summary of reported cases:	Number (%)	
**Total**	2846 (100.00)	4026 (100.00)
**Early stage**	1077 (37.84)	1077 (26.75)
**Late stage**	1395 (49.02)	2106 (52.31)
**No stage**	374 (13.14)	843 (20.94)
**DALY model:**		
**Mean age**	30.25	30.16
**Total deaths**	150.81 (5.3% mortality)	86.31 (2.1% mortality)
**YLDs**	451.7	3894.2
**YLLs**	3641.7	1891.6
**DALYs (age-weighted)**	4158.6	5785.8
**DALYs (without age-weighting)**	3407.8	4747.4

### Years of Life Lived with Disability (YLDs)

The haemolympathic early stage of HAT manifests in intermittent fever, headache, swollen lymph nodes, pruritus, skin lesions, and edema, among other nonspecific symptoms. Late-stage or meningoencephalitic HAT is characterised by invasion of the central nervous system, with symptoms including disruptions to the sleep cycle, neuropsychiatric, and endocrinal disorders [11], [38]. Overall, clinical symptoms are similar for *rhodesiense* and *gambiense* HAT, though the duration and progress of symptoms differ markedly; *rhodesiense* progresses from early to late stage and death typically within weeks to months, while *gambiense* HAT may progress over many months to years, with a more chronic course of infection [11], [39], [40]. Disability quantification is thus stage-specific, with late-stage illness implying substantively higher disability than early-stage illness.

YLDs were calculated for all cases, assuming that all fatal cases passed through a period of early- and late-stage disability before death. Disability weightings of 0.21 for early-stage illness and 0.81 for late-stage illness were used for *rhodesiense* HAT as per Fèvre et al. [25]. Stage-specific disability weightings have not been estimated for *gambiense* HAT. Weightings of 0.191 for early-stage illness and 0.81 for late-stage illness were thus used, based on estimates in the GBD study [22] and published estimates for *rhodesiense* HAT, respectively. These estimates reflect broadly similar clinical features for the two parasites despite different rates of progression, although some differences exist, with *gambiense* HAT generally being clinically milder and more variable [11]. The average duration of illness was estimated as 6 months for *rhodesiense* HAT, with 3 months of early stage and 3 months of late stage illness. This was based on various published estimates: 21 days for early-stage illness and 61 days for late-stage illness pre-admission [41], a median survival time of 4 months in the early 1900s epidemic in Uganda [42], 6 months average total duration [39] and 2–3 months late-stage duration in patients from Tanzania and Uganda [43]. The average duration for *gambiense* HAT was estimated to be 2 years for early-stage illness and 1 year for late-stage illness, based on estimates of 36 months early-stage and 12 months late-stage by Lutumba et al. [24] and 526 days early-stage and 500 days late-stage by Checchi et al. [40]. Burden calculation was done regardless of the point of diagnosis, in order to account retroactively for HAT impact prior to treatment. Cases were assumed to present at the beginning of early- or late-stage illness. Thus, YLD calculations included pre-diagnosis morbidity.

### Years of Life Lost (YLLs)

Age-specific mortality was modelled using a quadratic function, with average mortality adjusted based on parasite type and disease stage (see Figure S3). Average mortality was estimated at 2.4% (early stage) and 8.1% (late stage) for *rhodesiense* HAT [36]. This is consistent with estimated case fatality rates of 8.4–9.3% for melarsoprol-treated late-stage *rhodesiense* HAT in Tanzania and Uganda [43]. Treatment-based case-fatality ratios (CFRs) have been estimated for late-stage *gambiense* HAT cases in the late 1990s and early 2000s [44]. Based on this, an average mortality rate of 3% was used for late- stage *gambiense* HAT. Most cases of *gambiense* HAT in northwestern Uganda prior to the mid-2000s were treated with melarsoprol, with a mortality rate of 5–5.9% [44], [45]. However, Uganda changed its first-line treatment from melarsoprol to eflornithine (1.2% mortality) treatment in 2002 [44], [46]. Furthermore, the newer nifurtimox-eflornithine combination therapy was tested in various sites in Uganda during the study period [47], and shows promise as a lower-mortality treatment regimen for late-stage *gambiense* HAT [16], [48]. Thus, a reduced late-stage mortality rate of 3% was used as a conservative estimate. Negligible published data on early-stage mortality for *gambiense* HAT exist; a figure of 1% was used as an estimate since the mortality for early-stage *gambiense* illness is known to be low, as treatment-related complications due to pentamidine administration rarely occur [11], [38].

These stage- and parasite- adjusted mortality distributions were used to assign the age-specific probability of death in each iteration of the DALY model. YLLs were calculated only for those cases that were randomly assigned as deaths, based on the age-specific mortality probability.

### Unreported DALYs

We use the term ‘under-reporting’ herein to refer to the proportion of the estimated total number of cases which are not detected by active or passive screening. This lack of detection of HAT cases is known to be significant, yet remains poorly estimated. For *rhodesiense* HAT, a model of under-reporting based on the early to late stage ratio of presenting cases has estimated that approximately 40% of cases go unreported – and die – in Uganda [21], [49]. For *gambiense* HAT, the level of under-reporting has not been directly estimated. Robays et al. [20] estimated the effectiveness of active case-finding and treatment in the DRC at less than 50%, and Lutumba et al. [24] subsequently used this work to estimate that 40% of *gambiense* HAT cases in a rural community of the DRC went undetected following one round of active screening. Burden estimates are limited by the need to properly quantify HAT under-reporting [19], particularly as disease surveillance is lacking in many HAT-endemic areas [50], [51]. Thus, while an under-reporting level of 40% was used when DALYs were estimated and mapped by parish, we conducted a sensitivity analysis, varying the reporting rate to account for the significant uncertainty regarding levels of HAT under-reporting.

In the sensitivity analysis, the rate of under-reporting was varied in order to determine the effect on the magnitude and distribution of burden. For each reported case, the number of cases unreported was estimated based on various proportions of unreported cases: 0%, 20%, 40% for both parasites, as well as 67% (*gambiense*) and 95% (*rhodesiense*) based on the 2004 GBD study [22]. As described above, 40% was taken as a conservative estimate of the actual under-reporting rate when summing and mapping DALY totals by parish. All unreported *rhodesiense* HAT cases were assumed to be untreated and 100% fatal. Unreported *gambiense* HAT cases were assumed to be 50% fatal. The average duration of a *gambiense* HAT case is 3 years [40], and cases of *gambiense* HAT which remain undetected following one active screening round could in practice be detected in subsequent active screening rounds or through passive detection [24]. Because of the long duration of illness and possibility of subsequent detection, the 50% mortality estimate for unreported *gambiense* HAT conservatively accounts for the likelihood that a significant proportion of undetected cases may not in fact lead to fatality. Thus, early- and late-stage YLDs were calculated for all unreported cases, and YLLs were calculated for all unreported *rhodesiense* and 50% of unreported *gambiense* cases. We assumed that all fatal unreported cases passed through the full disease duration before death. Case age for unreported cases was assigned independently of the reported case ages, using the late-stage age distributions and methods described above.

### Burden comparison and mapping

Geographic location in latitude and longitude coordinates was available in the WHO dataset, with the exclusion of 78 *rhodesiense* cases and 23 *gambiense* cases which were missing latitude/longitude values. Furthermore, 8 *rhodesiense* HAT cases were identified as exported, as they represented patients who had migrated outside of HAT transmission zones. These were excluded to restrict the analysis to areas of active HAT transmission. These exclusions resulted in a total of 2760 *rhodesiense* and 4003 *gambiense* HAT cases which were mapped and analysed in ArcGIS (ESRI version 10). DALY totals for 2000–2009 were mapped and summarised by parish, the smallest administrative unit in Uganda. District population data were obtained from the 2002 Uganda census and projected to 2009 in the UN COD-FOD Registry [52]. Population values were averaged for the period 2002–2007 to give estimates representative of the study period.

DALY totals were averaged over 2000–2009, and divided by district population data to give values in terms of DALYs per 100 000 persons per year. This enabled comparison of the relative burden across districts with the national burden rates for HAT and other infectious diseases in Uganda, based on the DALY rates published in the 2004 GBD study [22].

## Results

### Burden of reported cases

Table 2 summarises the total burden attributable to reported HAT cases in Uganda, summed over the period 2000–2009. Results estimated a total of 4159 reported DALYs for *rhodesiense* HAT in southeastern Uganda from 2000–2009 (an average of 1.46 DALYs per case). The majority (88%) of the burden resulting from reported cases was due to years of life lost, with 12% of the burden due to years of life lived with disability. The total burden for *gambiense* HAT in northwestern Uganda was estimated at 5786 reported DALYs (an average of 1.44 DALYs per case). In contrast to *rhodesiense*, the majority (67%) of the burden of *gambiense* HAT resulted from years of life lived with disability. This is attributable to the longer duration and lower mortality rates of chronic g*ambiense* HAT, whereas the acute, high-mortality *rhodesiense* form tends to progress more quickly and results in a greater burden due to mortality.

### Sensitivity to under-reporting

As shown in Table 3, model results are highly sensitive to reporting rate, particularly for *rhodesiense* HAT. When under-reporting was accounted for, unreported DALYs formed the bulk of the total burden. Total burden for unreported cases was high for both forms of HAT, with overall burden being higher for *gambiense HAT*. DALY totals for *rhodesiense* HAT for 2000–2009 ranged from less than 4200 DALYs with 0% of cases unreported, to approximately 54 000 DALYs with 40% of cases unreported and well over 1 million DALYs with 95% of cases unreported. For *gambiense* HAT, total DALYs ranged from less than 5800 DALYs with 0% under-reporting, to approximately 44 000 DALYs with 40% under-reporting and 123 000 DALYs with 67% under-reporting. The average annual DALY total when 40% of cases were assumed as unreported was 9 814 DALYs, resulting from an estimated average of 1 145 cases per year. This is significantly lower – approximately one-quarter – when compared to the existing GBD estimate of 59 423 DALYs. When GBD estimates of under-reporting were used, however, the annual average burden was more than double the GBD 2004 estimate at 136 287 DALYs, resulting from an estimated total incidence of 6 912 cases per year. Thus, the bulk of this burden resulted from unreported mortality of *rhodesiense* HAT.

**Table 3:** Total HAT burden in Uganda for 2000–2009 in age-weighted DALYs, with sensitivity analysis of the under-reporting rate.

	*Rhodesiense* HAT		*Gambiense* HAT		Total HAT
Under-reporting rate:	Cases	DALYs	Cases	DALYs	DALYs
**0%**	2 846	4 159	4 026	5 786	9 944
**20%**	3 558	22 803	5 033	20 222	43 025
**40%**	4 743	53 828	6 710	44 313	98 141
**Annual average**					9 814
**GBD 2004 under-reporting rates:**					
**67%**	–	–	12 200	123 199	
**95%**	56 920	1 239 672	–	–	
**Total**					1 362 871
**Annual average**					136 287
**GBD 2004 estimate**					59 423

### Spatial and temporal variation in burden

**Figure 2:**
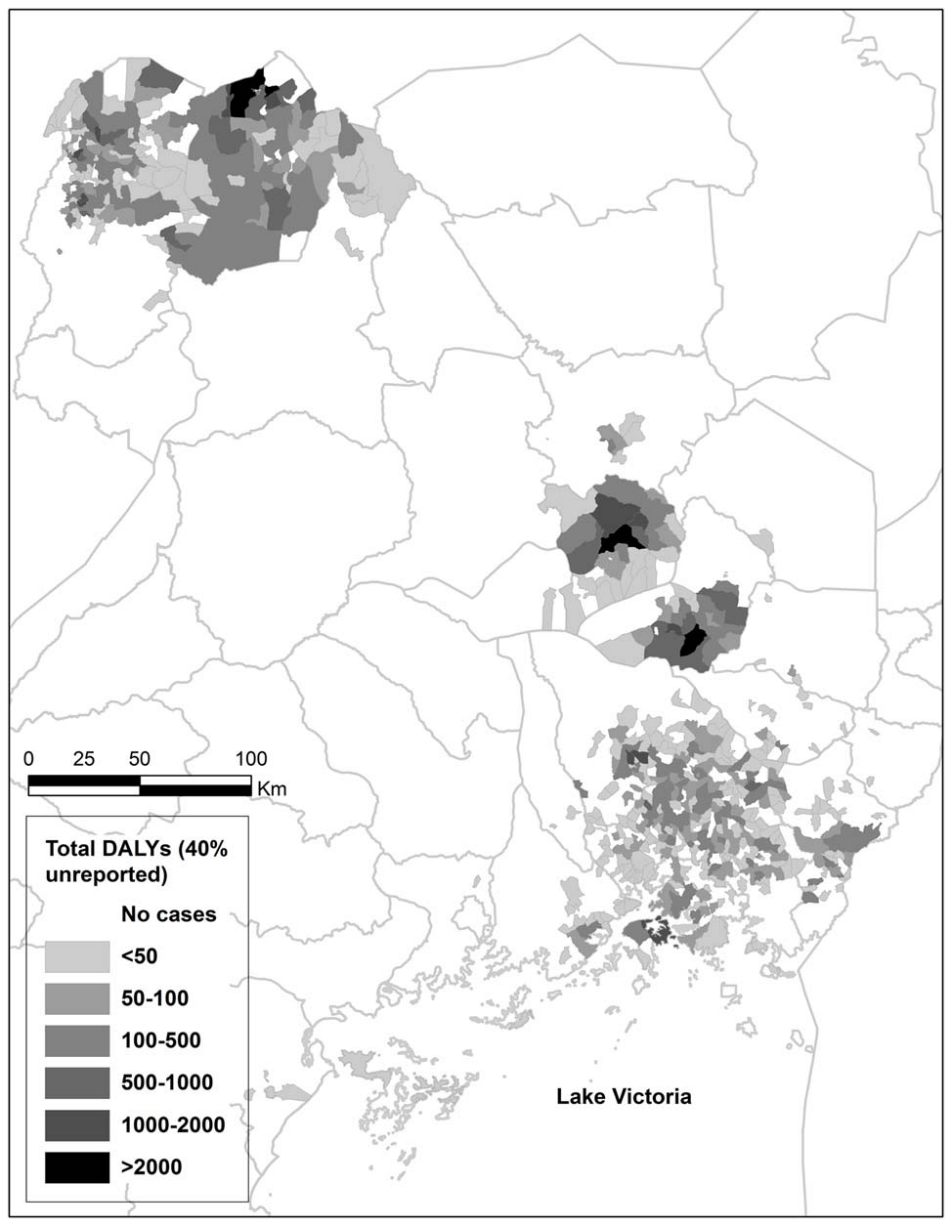
Total HAT burden in Uganda by parish, 2000–2009. Shaded parishes indicate the total burden in DALYs estimated over the ten-year study period, as modelled with an under-reporting rate of 40% [21].

Spatial variation in burden was significant for both forms of HAT, with cases highly focally concentrated in a small number of parishes. This is shown by the parish-level burden map in Figure 2. In the *rhodesiense*-endemic areas of central and southeastern Uganda, 413 parishes in total were affected by *rhodesiense* HAT. 62% of parishes had fewer than 5 reported DALYs, while 5 parishes (1.2%) were afflicted by over 100 DALYs, and these 5 parishes accounted for 21.3% of the burden of reported cases. In northwestern Uganda, 189 parishes were affected by *gambiense* HAT. 25% of parishes had less than 5 reported DALYs, while 12 parishes (6.3%) were afflicted by over 100 reported DALYs, accounting for 39.4% of the burden of reported cases. Similarly, when total burden was calculated with 40% under-reporting, 2 parishes in southeastern Uganda, Alwa and Kateta, accounted for over 2000 DALYs each, forming over 9% of the total burden. In northwestern Uganda, 2 parishes accounted for over 2000 DALYs each. A large portion of the burden of g*ambiense* HAT was concentrated in a few severely afflicted parishes, whereas the burden of *rhodesiense* HAT was more geographically widespread across a greater number of parishes.

**Figure 3:**
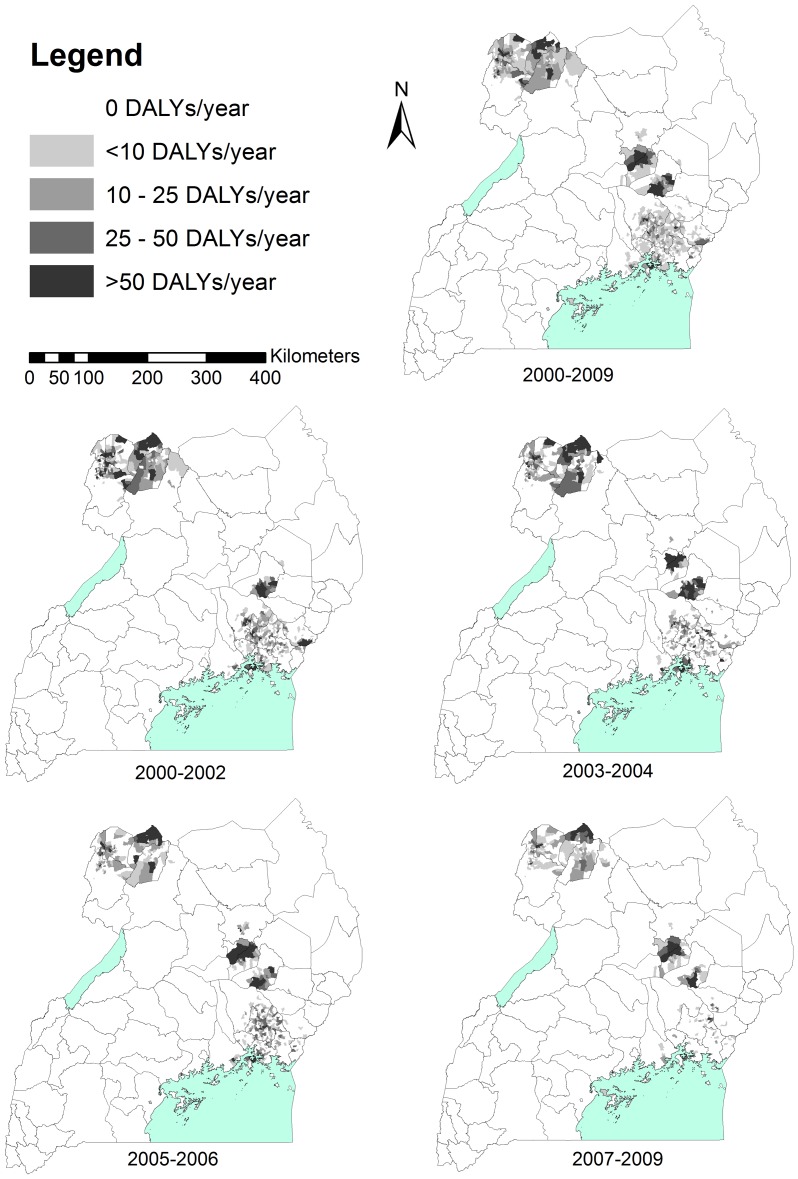
Temporal variation in HAT burden by parish, 2000–2009. Total DALYs per year, as modelled with an under-reporting rate of 40%, are shown as averaged over the 2000–2009 historical period, and four sub-periods. DALYs/year were calculated and mapped by parish, and district boundaries are shown.

Temporal variation in burden over the 2000–2009 period also showed an overall decreasing trend, reflective of the decrease in overall HAT incidence. These temporal trends varied by parish and district, as shown in Figure 3 and Table 4. The most highly affected areas varied over time. For instance, in the district of Kaberamaido in central Uganda, foci of HAT burden did not occur until 2003, showed a peak in the middle of the time period, and burden was decreasing as of the late 2000s. On the other hand, districts such as Iganga and Bugiri in southeastern Uganda showed a marked decrease in HAT burden over the same ten-year time period.

**Table 4:** DALYs per year by district, showing temporal trends in HAT burden calculated with a 40% under-reporting rate, averaged over 2000–2009 and four sub-periods.

	DALYs/year				
District	2000–2009	2000–2002	2003–2004	2005–2006	2007–2009
Adjumani	403.4	628.8	560.6	231.6	187.7
Amuru	40.1	45.4	74.2	5.5	35.1
Arua	1604.6	2884.4	1612.0	1191.0	595.6
Bugiri	239.4	308.6	277.1	351.6	70.2
Busia	122.0	102.0	306.7	101.7	32.4
Dokolo	291.1	0.0	155.9	556.1	495.5
Iganga	1073.7	1604.7	1014.0	1670.9	184.4
Jinja	70.8	103.8	105.3	73.5	13.1
Kaberamaido	821.3	0.0	1154.0	1609.1	895.5
Kalangala	5.6	0.0	0.0	18.0	6.6
Kamuli	608.6	1468.3	569.7	243.4	18.3
Katakwi	2.0	6.8	0.0	0.0	0.0
Kayunga	5.8	7.0	18.4	0.0	0.0
Kumi	11.6	6.2	29.2	19.6	0.0
Lira	39.9	0.0	19.1	141.2	26.0
Mayuge	193.3	296.6	244.2	249.3	18.7
Mbale	5.3	5.8	9.3	8.4	0.0
Moyo	1785.0	2431.3	2718.2	1644.0	610.5
Mukono	275.5	403.0	335.9	291.3	97.1
Pallisa	59.6	19.4	40.2	19.0	139.7
Soroti	1097.1	1275.6	1759.8	1190.5	414.6
Tororo	297.0	841.4	155.6	46.5	13.9
Wakiso	1.8	6.0	0.0	0.0	0.0
Yumbe	574.0	1145.7	436.6	340.1	249.8

**Figure 4:**
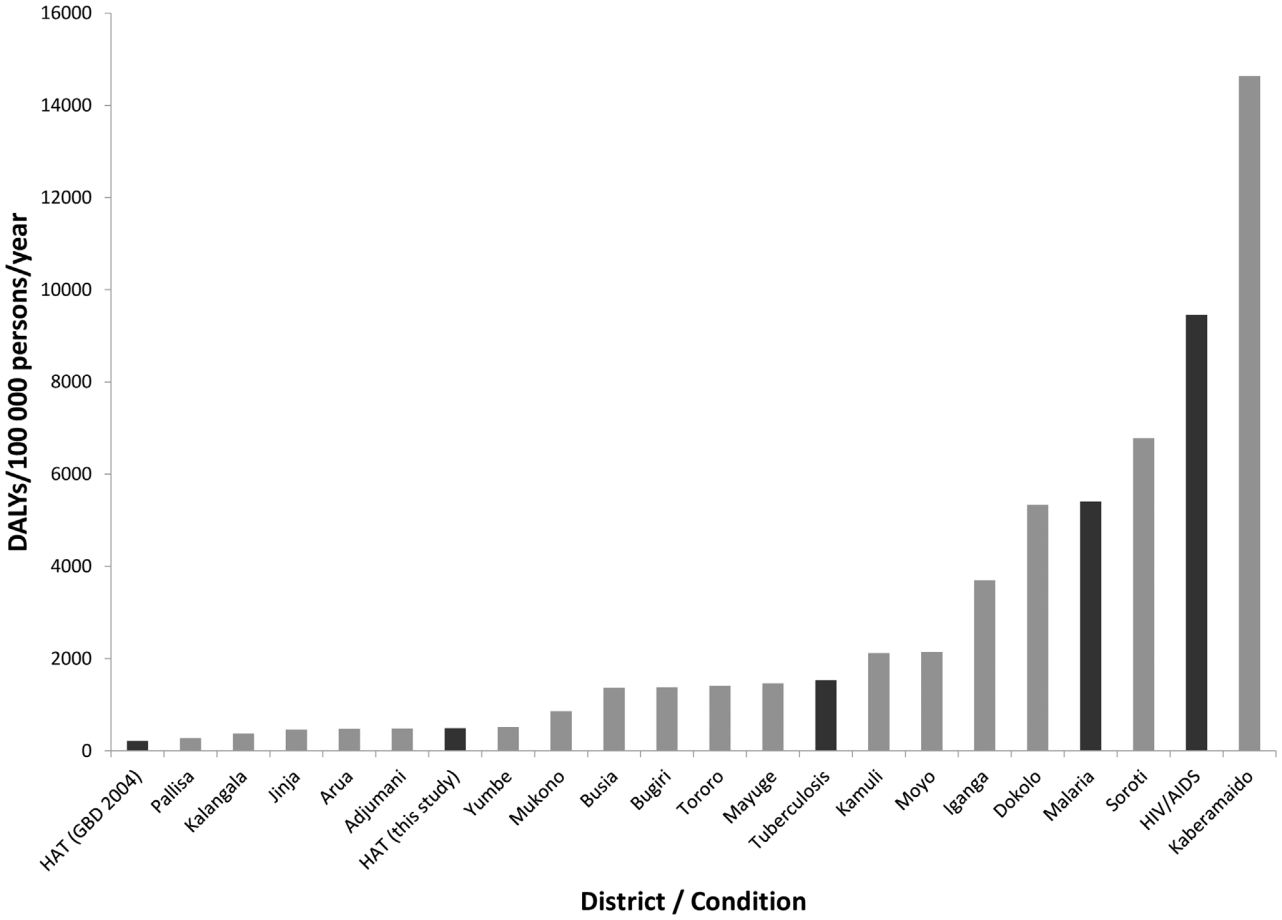
Comparison of average annual DALY rates for HAT and other infectious diseases. The calculated historic average annual DALY rates by district population over the period 2000–2009 are shown in grey. The calculated national average DALY rate for HAT and 2004 GBD DALY rates for HAT and major infectious diseases in Uganda are shown in black. Estimates of completeness of case reporting for HAT (33% *gambiense*, 5% *rhodesiense*) and DALY rates for other diseases are taken from the GBD 2004 report [22].

Figure 4 shows that while 8 out of 25 affected districts (32%) had a lower rate of HAT burden than the national GBD estimate of 212 DALYs/100 000 persons/year, 6 districts had a burden rate comparable to the national burden rate for tuberculosis, 3 districts had a burden rate comparable to malaria, and one district had a burden rate larger than the national average for HIV/AIDS. We emphasise that the annual average 2000–2009 burden values shown in Figure 3 reflect past incidence. Given the declining trend in both forms of HAT incidence in Uganda, current annual burden rates are likely to be lower than shown. These results must be further qualified, by the fact that district burden rates for tuberculosis, malaria, and HIV/AIDS, which have not been estimated, would likely exceed national averages in highly affected areas, some of which could overlap with HAT-endemic regions.

The most highly afflicted districts, Kaberamaido and Soroti, were located in the northern area of the *rhodesiense* HAT transmission zone in central Uganda. Kaberamaido had a lower annual average DALY total (21 381) than Soroti (28 475) and Iganga (28 404), but also represented a smaller district population. Dokolo and Iganga, also *rhodesiense* HAT-endemic districts in the southeast, had the 3^rd^ and 4^th^ highest DALY rates, respectively. Moyo was the *gambiense* HAT-endemic district with the highest DALY rate and 5^th^ overall, with an annual average total of 5 048 DALYs and a DALY rate of 2 144 DALYs/100 000 persons/year. Notably, district DALY rates do not account for within-district spatial variation in burden. As discussed above and demonstrated in Figures 2 and 3, burden varies significantly across parishes. Thus, parish-level per capita burden would be higher than district DALY rates in the most highly affected parishes.

## Discussion

Our results encompass new HAT burden estimates for Uganda , incorporating revised parameters reflecting the stage- and parasite-specific determinants of burden. They highlight thehigh sensitivity to under-reporting, and sub-national spatial variation in burden, which is inadequately captured by global burden estimates. Mortality was the main contributor to DALY burden for *rhodesiense* HAT but morbidity the main component of *gambiense* HAT burden. This is due to the distinct clinical features of the two stages of disease morbidity, as well as the differential durations and mortality rates for the two forms of the disease. While the GBD 2004 update distinguishes between disease durations, it does not distinguish stage-specific disability weightings for either form, nor does it account for the differences in mortality rates between the two forms of HAT. Moreover, certain parishes and districts in Uganda were found to bear an extremely heavy burden due to HAT morbidity and mortality. Critically, this focality in burden has not been accounted for by past national burden estimates.

The significant spatial variation in DALY burden across parishes demonstrates the value of evaluating the burden of focalised diseases such as HAT on a sub-national scale. While the national total burden of HAT was low relative to other conditions, highly-affected districts in Uganda, particularly Kaberamaido, Soroti, Dokolo, and Iganga in the southeast, and Moyo in the northwest, had per capita burden levels comparable to or higher than HIV, malaria, and tuberculosis. It is important to note, however, that the sub-national burdens of these diseases could also vary across geographic areas and sub-populations. Hence, similar spatial burden rate evaluation for other conditions, besides HAT, is merited for a district-level comparison. Nonetheless, comparison with national burden rates shows that HAT burden operates on a local level at a scale similar to the estimated and acknowledged high impacts of HIV, malaria, and tuberculosis in the country as a whole. Cost-benefit analysis of intervention in these highly affected regions may be particularly favourable for focal diseases when local burden and geographically-targeted interventions are taken into account.

We note several potential sources of bias and limitations to this study.

### Temporal variation

HAT is not only spatially but also temporally focal, occurring in epidemics of high transmission and incidence [11]. We have included estimates of parish- and district-level burden sub-divided into total annual burden for periods of two to three years, but note that other results presented herein are estimated totals and average yearly values for 2000–2009, which do not reflect the overall change in HAT incidence over time or the variation in incidence from year to year. In order to reflect the current burden situation in the affected districts of Uganda, updated spatial data on HAT incidence would be necessary. In addition, temporal variation in HAT would imply burden rates higher than those presented here since disease burden will be concentrated during epidemic years. Both spatial and temporal variation in HAT burden show the concentration of disease impact in those communities bearing the brunt of the burden of focal HAT epidemics.Accordingly, the potential benefits of targeted interventions in these areas will also be greater..

### Spatial variation

We have used case incidence data to map the burden of HAT in Uganda at a spatially specific sub-national scale. However, data on spatial variation in other model parameters, such as the age of incidence, mortality, or under-reporting rates (discussed below), were not available. Hence, the spatial variation in burden identified reflects that of incidence. Nonetheless, this method to derive HAT burden values from incidence allows DALYs to be compared across parishes or districts, or with other conditions at varying spatial scales. Burden units enable this comparison, which would not be possible from examining disease incidence alone.

### Under-reporting

Under-reporting of fatal cases has been noted previously as an important contributor to the DALY burden of HAT, but has not been quantified adequately in burden calculations [19]. When estimates of the proportion of unreported cases were included, there was a greater than 4-fold increase in burden with 20% under-reporting, and a nearly 10-fold increase in burden with 40% under-reporting. These increases were greater for *rhodesiense* HAT due to 100% mortality of unreported cases, with a greater than 5-fold and nearly 13-fold increase in burden with 20% and 40% under-reporting, respectively. *Gambiense* HAT showed more modest increases of 3.5- and 7.7-fold when 20% and 40% under-reporting was accounted for. Notably, these results are likely conservative given that Uganda's national sleeping sickness program shifted in 2005 from active to passive surveillance, resulting in reduced reporting [36]. Thus, average under-reporting rates for the study period of 2000–2009 may have been even higher than estimated.

Under-reporting estimates have only been validated for *rhodesiense* HAT in southeastern Uganda, however, and may not be applicable for *gambiense* HAT in the northwest. Because of the milder and more varied clinical course of the natural progression of *gambiense* HAT, cases are typically detected through active case-finding. Though estimates of *gambiense* HAT under-reporting are lacking, surveillance activities have been shown to have poor sensitivity. Robays et al. [20] used a model to show that active case-finding and treatment failed to detect a significant portion of *gambiense* HAT cases, and Lutumba et al. [53] found that *gambiense* HAT screening failed to avoid more than 60% of deaths in the DRC.

Broad under-reporting estimates also inadequately account for spatial variation in under-reporting related to the presence of HAT treatment centres. As discussed above, data on spatial variance for input parameters such as the under-reporting rate would strengthen the findings of this study. The proportion of early-stage patients detected was shown to be inversely related to distance from health unit by Odiit et al. [54]. However, it is difficult to define the causation between reported case incidence and the presence of treatment centres, as facilities are often deployed in response to sleeping sickness outbreaks.

Efforts to quantify DALY burden due to HAT in Uganda remain constrained by a lack of robust local estimates of HAT under-reporting. The importance of improved and sustained disease surveillance efforts, notwithstanding recent reductions in reported HAT incidence, has been widely emphasized in order to promote control and elimination of HAT in endemic areas [16], [30], [55], [56]. This study demonstrates their further importance in order to better account for the under-reported burden of HAT.

### Disease duration

Broad duration estimates were used for each stage and form of the disease. This could overestimate duration for patients diagnosed and treated early in the disease progression. However, significant delays (median 7 months) in patient presentation and diagnosis for *gambiense* HAT have been observed [57], which underscores the improbability of early case detection and treatment. Significant variability in disease duration can exist, particularly in the case of *gambiense* HAT [58], while varied disease severity and progression rates between foci have been observed for *rhodesiense* HAT in Uganda [59].

Furthermore, YLDs were calculated for all fatal as well as non-fatal cases, assuming all fatal and all unreported cases passed through the full early- and late-stage duration. This may have resulted in an overestimate of burden if cases died in the early stage or did not progress through the full late-stage duration. However, our duration estimates were based on multiple published estimates and were shorter than the 1-year (*rhodesiense*) and 5-year (*gambiense*) estimates used by the GBD 2004 study [22], thus may be considered conservative.

### Mortality

Mortality estimates for *gambiense* HAT are highly dependent on the treatment used. Information on treatment and mortality were not included in the dataset, which limited the accuracy of the *gambiense* mortality estimates. Furthermore, the treatment regimen traditionally used for late-stage *gambiense* HAT is melarsoprol, and a high incidence of melarsoprol treatment failures for *gambiense* HAT has been recorded in Uganda [60]. However, lower-mortality eflornithine and combination therapy treatments were introduced and tested in Uganda throughout the study period [44], [46], [47]. Thus, lack of data on treatment and shifts in treatment regimens led us to conservatively estimate *gambiense* HAT mortality based on published case-fatality ratios.

In addition, 100% and 50% fatality of unreported cases was assumed for *rhodesiense* and *gambiense* HAT, respectively, with all unreported cases progressing through early and late stages. However, this would overestimate burden if cases spontaneously recovered or were trypanotolerant. Recent research has indicated the possibility of recovery for *gambiense* HAT in West Africa [61] but not *rhodesiense* HAT. However, previous research concluded that self-resolving and trypanotolerant *gambiense* HAT infections, if they do occur, are a small minority [23]. The possibility and frequency of non-fatal *gambiense* HAT occurrence remains unresolved, but seems unlikely to significantly impact HAT burden estimates.

### Burden parameterization

The GBD 2010 study was published in late 2012, and presented revised methods for DALY calculations. See Text S1 for a full discussion of the differences in burden parameters and their potential effect on the results of this study. Notwithstanding these changes, we identify key priorities for estimation of burden parameterization for HAT:

1. Disease and stage-specific disability weightings
  The use of a single disability weighting of 0.191 for HAT illness, without distinguishing between stages or forms of the parasite, represents a significant constraint to burden estimation. Late-stage HAT involves a severe neuropsychiatric disorder which imposes significant limitations on patients' activities, compared to the nonspecific symptoms of early-stage illness. Furthermore, the current disability weight does not reflect differences between *rhodesiense* and *gambiense* HAT.
  Disability weightings were re-evaluated for the GBD 2010 update using a combination of household and open-access online surveys [62]. However, the new weights derived have not accounted for the stage- and parasite-specific parameters of HAT infection. Instead, HAT has been grouped with other infectious diseases, with weight estimates of 0.005, 0.053, and 0.210 for mild, moderate, and severe acute episodes, respectively [63]. These disability weightings are therefore less specific to HAT and do not fully reflect the debilitating symptoms of the stages and types of HAT illness.
2. Duration of sequelae
  The use of broad estimates of 5-year duration for *gambiense* HAT and 1-year duration for *rhodesiense* HAT do not account for the differential durations of disease stages. In combination with stage-specific disability weightings, a breakdown of HAT illness duration by stage would provide for more accurate burden estimation.
3. Parasite- and treatment-specific mortality rates
  The GBD mortality estimates do not reflect differential mortality rates for *rhodesiense* and *gambiense* HAT and their associated treatments, but the two forms of HAT disease have markedly different clinical courses and outcomes. Mortality for the acute *rhodesiense* HAT and treatment with the melarsoprol regimen are higher than for *gambiense* HAT and the recently developed eflornithine or drug combination therapies [44], [46]. To accurately determine HAT burden, further data on the case fatality rates associated with particular treatment regimens and on the actual deployment of various treatments in the study area and time period of interest are required.
4. Spatially disaggregated burden
  Most importantly, national-level burden estimates, though useful tools for global assessment of relative disease burden, do not reflect the focalization of spatially heterogeneous disease incidence for infections such as HAT.

Limitations to burden parameterization are not unique to HAT. Parameters have been developed in varying levels of specificity for other diseases. For instance, GBD 2004 disability weightings for tuberculosis, malaria, and HIV/AIDS vary with age; weightings for malaria and HIV/AIDS also distinguish three separate disease sequelae and vary with treatment. Methods for estimating mortality rates vary across these three conditions, although none share the parasite- and treatment- specific issues related to HAT. While acknowledging that these varied assumptions underlie the DALY estimates used for broad comparison with other diseases, an in-depth evaluation of the GBD parameters for these diseases is beyond the scope of this study. It is critical that the public health research community move beyond the GBD framework's focus on nationally aggregated burden to consider spatial – and temporal – variation in burden. This is particularly relevant for neglected tropical diseases whose current burden values are already poorly estimated [5], [7]. While national estimates remain a useful tool for systematic comparison of global trends, their use for prioritization of health intervention and funding has failed to consider the scale dependence of disease burden. While absolute DALY burden may be relatively low for focal diseases, the cost-benefit ratio of intervention measures targeted at small areas of high burden may be favourable compared to diseases with geographically dispersed burden. Sub-national burden evaluation for a range of conditions may allow quantitative comparison of the spatial variation in burden across diseases. This would also allow identification of those areas and populations suffering from highly focalized impacts and hence the greatest potential benefits of targeted interventions. This is especially imperative since we suspect HAT is not the only disease that shows a focalized burden, and further, multiple diseases may impact the same local populations and merit more general public health practices or combined interventions. Burden estimates are established as a valuable guide to directing scarce disease control resources, yet in many cases disease surveillance and health policy interventions operate at local levels. In compliment to the recent methodological revisions to the GBD study, it is thus imperative that the use of the DALY also be revised to validate and test its utility at differential spatial scales.

## Supporting Information

Figure S1 **Probability distribution for early-stage case age.** A negative binomial function was fit to the age of 942 early-stage HAT cases [36] using the *nbfit* function in STATA 11 software. Parameters (standard error): p = 0.92958 (0.00351), r = 2.45125 (0.12014), μ = 32.35775 (0.69842). This probability distribution was used to assign age to early-stage cases from the WHO Atlas of HAT dataset when modelling DALY burden.
(TIF)

Figure S2 **Probability distribution for late-stage case age.** A negative binomial function was fit to the age of 1426 late-stage HAT cases [36] using the *nbfit* function in STATA 11 software. Parameters (standard error): p = 0.92535 (0.003), r = 2.4361 (0.09678), μ = 30.19705 (0.5326). This probability distribution was used to assign age to late-stage cases from the WHO Atlas of HAT dataset when modelling DALY burden.
(TIF)

Figure S3 **Probability distribution for** ***rhodesiense*** **HAT mortality.** A quadratic function was fit to mortality rates for *rhodesiense* HAT cases taken from Berrang Ford et al. [36] using the *qfit* function in STATA 11 software. Parameters: a = 0.0000248, b = −0.0016197, c = 0.0757683. Average mortality, weighted by incidence = 0.058686.
(TIF)

Table S1 **Uganda-specific life tables.** 2000 and 2009 life expectancy values were averaged and weighted by the sex ratio shown in HAT case incidence, with 51.6% of cases being male [36].
(DOC)

Text S1 **GBD 2010 revisions.**
(DOCX)
