# Supplementary material for: Incorporating Scale Dependence in Disease Burden Estimates: The Case of Human African Trypanosomiasis in Uganda
Source: PLoS Negl Trop Dis. 2014 Feb 13;8(2):e2704. doi: 10.1371/journal.pntd.0002704 (PMC3923749; doi:10.1371/journal.pntd.0002704)
Supplement: Table S1 — Uganda-specific life tables. 2000 and 2009 life expectancy values were averaged and weighted by the sex ratio shown in HAT case incidence, with 51.6% of cases being male [36]. (DOC) [file pntd.0002704.s004.doc]

|  | Expectation of life at age x | | | |
| --- | --- | --- | --- | --- |
| Age group | 2000-2009 average, male | 2000-2009 average, female | 2000-2009 average, both sexes | 2000-2009 average, sex-weighted |
| <1 | 45.65 | 53.50 | 49.30 | 49.45 |
| 1-4 | 49.50 | 56.85 | 52.95 | 53.06 |
| 5-9 | 48.75 | 56.05 | 52.15 | 52.28 |
| 10-14 | 44.65 | 51.75 | 47.95 | 48.09 |
| 15-19 | 40.20 | 47.15 | 43.45 | 43.56 |
| 20-24 | 35.80 | 42.55 | 38.95 | 39.07 |
| 25-29 | 31.90 | 38.40 | 34.95 | 35.05 |
| 30-34 | 28.35 | 35.05 | 31.50 | 31.59 |
| 35-39 | 25.30 | 32.20 | 28.50 | 28.64 |
| 40-44 | 22.75 | 29.45 | 25.90 | 25.99 |
| 45-49 | 20.40 | 26.50 | 23.35 | 23.35 |
| 50-54 | 18.00 | 23.25 | 20.60 | 20.54 |
| 55-59 | 15.60 | 19.80 | 17.65 | 17.63 |
| 60-64 | 13.15 | 16.35 | 14.75 | 14.70 |
| 65-69 | 10.75 | 13.05 | 11.90 | 11.86 |
| 70-74 | 8.50 | 10.10 | 9.35 | 9.27 |
| 75-79 | 6.60 | 7.65 | 7.15 | 7.11 |
| 80-84 | 5.00 | 5.75 | 5.40 | 5.36 |
| 85-89 | 3.80 | 4.25 | 4.05 | 4.02 |
| 90-94 | 2.80 | 3.10 | 3.00 | 2.95 |
| 95-99 | 2.10 | 2.30 | 2.20 | 2.20 |
| 100+ | 1.65 | 1.70 | 1.70 | 1.67 |
