# Supplementary material for: Incorporating Scale Dependence in Disease Burden Estimates: The Case of Human African Trypanosomiasis in Uganda
Source: PLoS Negl Trop Dis. 2014 Feb 13;8(2):e2704. doi: 10.1371/journal.pntd.0002704 (PMC3923749; doi:10.1371/journal.pntd.0002704)
Supplement: Text S1 — GBD 2010 revisions. (DOCX) [file pntd.0002704.s005.docx]

The GBD 2010 parameters, published after this study’s analysis was conducted, differ in four key ways from the pre-GBD calculations which the spatial HAT estimates are based upon:

First, the standard life expectancy at birth was revised upwards to 86.0 years for males and females, from 82.5 years for females and 80.0 years for males [[31](#_ENREF_31)]. Because Uganda-specific life tables were used for the HAT burden estimation (see Table 1 and Supporting Information Figure 1), this parameter change would not affect our burden estimates themselves. It would cause them to reflect an even more conservative estimate of burden, by increasing the gap between the Uganda-specific and standard life expectancies used for YLL calculations. It is important to note that life expectancy tends to increase over time, as noted in Table 1, so the rate of change of life expectancy in Uganda will determine the extent to which older life expectancy values cause burden underestimation.

Second, the GBD 2010 study accounted for comorbidity in YLD calculations, and computed YLDs as the prevalence of sequelae multiplied by the comorbidity-adjusted disability weight [[31](#_ENREF_31)]. This could cause our results to reflect an overestimate of burden, vis-à-vis the revised parameters, since some DALYs which are taken as attributable to HAT alone would affect individuals for which morbidity is an outcome of one or more other causes.

Third, the GBD 2010 abandoned the use of time discounting of DALYs [**31**]. This would cause the revised burden to be much higher than our estimates and those of previous GBD studies, which employed discounting. Thus, our burden estimates are much lower than they would be if the revised parameters were employed.

Fourth, the GBD 2010 abandoned the use of age weighting in DALY calculations [**31**]. We ran the model for HAT burden of reported cases without age-weighting, and found that the estimated burden was reduced by approximately 18% relative to the age-weighted total. This would reflect that non-age weighted DALYs emphasise infant and child deaths, whereas years of young and middle-aged adulthood, are given emphasis when age-weighting is employed. The age distribution of Ugandan cases in our model favoured these age ranges, with distribution means from 30-32 years (see Supporting Information Figures 1 and 2).
